# Bronisława Fejgin (1883–1943): Forgotten Important Contributor to International Microbiology and Phage Therapy

**DOI:** 10.3390/antibiotics10111353

**Published:** 2021-11-05

**Authors:** Andrzej Grzybowski, Maciej Żaczek, Andrzej Górski, Beata Weber-Dąbrowska, Ryszard Międzybrodzki

**Affiliations:** 1 Institute for Research in Ophthalmology, 60-836 Poznań, Poland; ae.grzybowski@gmail.com; 2 Department of Ophthalmology, University of Warmia and Mazury, 10-561 Olsztyn, Poland; 3 Bacteriophage Laboratory, Department of Phage Therapy, Ludwik Hirszfeld Institute of Immunology and Experimental Therapy, Polish Academy of Sciences, 53-114 Wrocław, Poland; andrzej.gorski@hirszfeld.pl (A.G.); beata.weber-dabrowska@hirszfeld.pl (B.W.-D.); ryszard.miedzybrodzki@hirszfeld.pl (R.M.); 4 Phage Therapy Unit, Ludwik Hirszfeld Institute of Immunology and Experimental Therapy, Polish Academy of Sciences, 53-114 Wrocław, Poland; 5 Infant Jesus Teaching Hospital, Medical University of Warsaw, 02-005 Warsaw, Poland; 6 Department of Clinical Immunology, Transplantation Institute, Medical University of Warsaw, 02-006 Warsaw, Poland

**Keywords:** phage therapy, antibiotic resistance, history of medicine, microbiology, bacterial viruses, d’Herelle phenomenon, bacterial lysis

## Abstract

Bronisława Brandla Fejgin was a Polish-born Jewish female physician. Among Fejgin’s numerous articles in the field of microbiology, her later work was almost entirely devoted to phage research. Although not equally famous as the phage pioneers from Western Europe, F.W. Twort and F. d’Herelle, Fejgin’s contribution to phage research deserves proper recognition. Her studies on phages resulted in the publication of numerous original scientific reports. These articles, published mostly in French, constitute an important source of information and expertise on early attempts towards therapeutic use of phages in humans. The interwar period marks the most intense years in Bronisława Fejgin’s research activity, brutally interrupted by her death in the Warsaw Ghetto in 1943. Her microbiology contributions have not been analyzed so far. Thus, the aim of this article is to fill the existing gap in the history of microbiology and phage therapy.

## 1. Introduction

**Figure 1:**
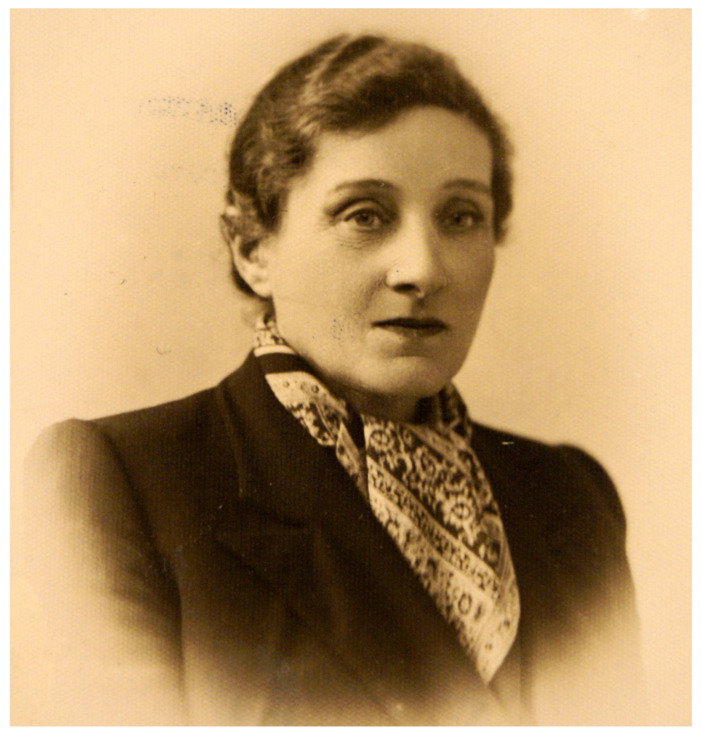
Bronisława Brandla Fejgin (1883–1943). Source: Main Physicians Library, Warsaw, Poland (public domain).

Frederick William Twort and Felix d’Herelle are considered to be the discoverers of phages and fathers of phage therapy [1]. However, one of the significant early contributors to this field, Bronisława Fejgin (Figure 1), who along with the aforementioned phage discoverers, conducted pioneering research on bacterial viruses and their therapeutic applications long before World War II, is largely forgotten.

There has been no scientific analysis of her works conducted in the literature. Thus, the aim of this study was to perform such an analysis supplemented with some reliable biographical information.

## 2. Methodology

We collected all of Fejgin’s available publications in Polish as well as an international database and bibliography encyclopedia (such as Main Library of the University of Gdańsk, Digital Library of the Medical University of Warsaw and The National Library of Poland). Then, we selected the most important contributions within 17 articles, 16 in French and one in Polish, among which she was the first author of 15 articles. They were further analyzed in detail, paying attention to scientific relevance, evidence of international cooperation and accuracy in drawing hypotheses.

## 3. Biography

**Figure 2:**
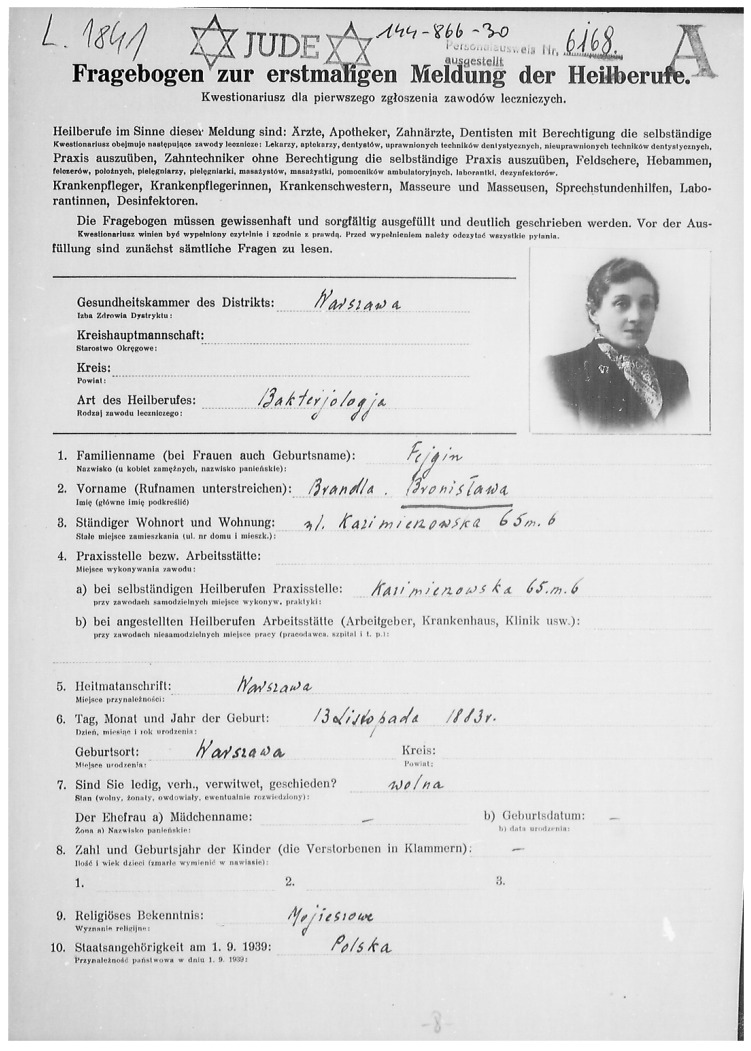
Dr. Bronislawa Fejgin’s Warsaw Ghetto Registration Document. Source: Main Physicians Library, Warsaw, Poland (public domain).

**Figure 3:**
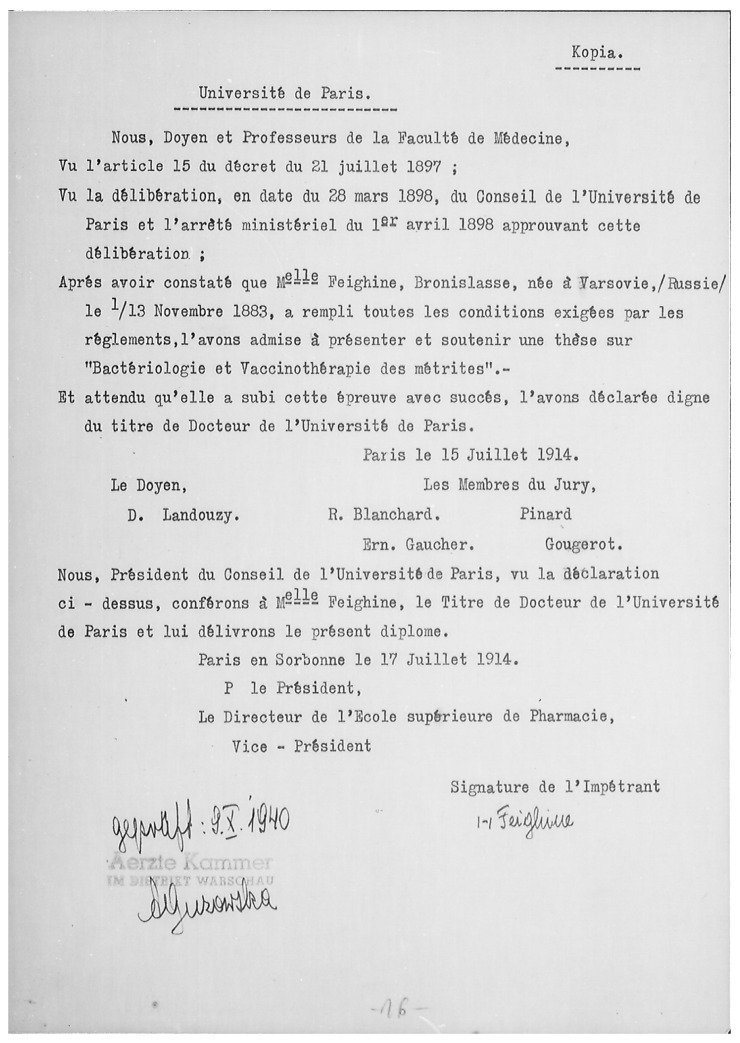
Copy of Bronislawa Fejgin’s French diploma. Source: Main Physicians Library, Warsaw, Poland (public domain).

Bronisława Fejgin was born in Warsaw into a Jewish family on November 13th, 1883 (Figure 2). Her life was not meant to be spent entirely on Polish soil in the territory of the Russian Partition (it is worth mentioning that the Polish state remained divided under foreign occupation until 1918). She studied medicine in the years 1907–1914 at Sorbonne Medical School in Paris. In 1914, she graduated university and defended a doctoral dissertation entitled “Bacteriology and vaccine therapy of metritis” (Figure 3). She confirmed her university diploma at Tartu University (today’s Estonia) in 1915. During the years 1914–1918, she worked at the Bacteriology Department of the Russian Red Cross. From 1922, she started working at the National Epidemiology Institute, later known as the National Hygiene Institute in Warsaw, where she worked till 1939. During this period, she visited Prof. Rudolf Weigl in Lviv (today’s Ukraine) to learn techniques necessary for experimental studies on typhus. In addition, she also visited bacteriology centers in Denmark, Germany and France. She learnt BCG vaccine and tuberculin production in the Pasteur Institute [2]. She was a very productive researcher who published the majority of her papers in French, and some in Polish, English and German. In 1940, following the German invasion of Poland and establishment of the Warsaw Ghetto, the largest Nazi ghettos during World War II for segregating and confining Jews, she became the head of the bacteriology department of the Chemical-Bacteriological Institute and was the teacher of bacteriology and serology in the clandestine Medical School therein. She died in January 1943 during a mass murder in the Ghetto [3,4,5,6,7,8,9,10]. She was never married, nor did she have children.

## 4. Analysis of Scientific Contributions

The first article co-authored by Bronisława Fejgin was published as early as 1913, when she was still a medical student. As a result of cooperation with D. M. Bertrand, she focused on bacterial microflora and its role in uterine infections. The authors isolated and described in detail a bacteria-producing green pigment which was responsible for infections in female patients [11]. The authors were able to link the presence (or absence) of bacteria to the presence of uterine discharge. In the same year, both authors managed to prepare vaccines based on their bacterial isolates. Those vaccines were highly successful following repeated injections in treating uterine infections caused mostly by childbirth or miscarriage [12].

Although we want to emphasize Fejgin’s contribution to phage research, her earliest work clearly went beyond interest in phages. She focused on microbiology and serology without a clear distinction towards phages. In fact, she did not even mention them in several of her articles. For instance, she described a plant tumor that had grown on *Pelargonium zonatum* following inoculation of a bacterial strain isolated from human cervix carcinoma [13]. Today, we know that studying bacteria sooner or later would probably lead her to observe a spontaneously occurring “lytic agent” in the form of “virgin spots” as described by her in an article from 1924 based on observations of a 24-h *Proteus* culture [14]. Lysogeny has been adapted by numerous species of bacteria as a form of protection in the environment for phages, e.g., inactivation by UV irradiation and can be found as often as in half of all culturable marine environmental bacterial isolates [15]. Notably, Fejgin’s efforts to isolate and investigate the lysogenic strain of *Proteus* described in the aforementioned article from 1924 was appreciated decades later by Coetzee and Sacks in Nature [16].

The first known article of Bronisława Fejgin focusing on phages dates to 1923. Together with Janusz Supniewski, a Polish physician, pharmacologist, chemist and a Ph.D. student of Prof. Ludwik Hirszfeld, they devoted this work to “d’Herelle phenomenon”, as exactly provided in the title [17]. Once she noticed bacterial lysis, her attention turned to this phenomenon for years. In 1927, she predicted in a Polish medical journal that facts related to the discovery of “invisible bacterial forms” were ready to refute all knowledge on the morphology of microbes and shake faith in the stability of bacterial species [18]. In 1931, she authored an extensive, for those times, article on marine microbes published in the Bulletin de l’Institut Oceanographique [19]. One would be wrong to assume that the article was only devoted to the marine ecosystem. At the very beginning of the above article, in the second paragraph, Fejgin mentioned the phenomenon of d’Herelle bacteriophagy by describing it as one of the most important forms of acquired and natural immunity in living organisms. This fact is consistent with our earlier assumption that working on marine bacterial strains, among which lysogeny is a common occurrence, would inevitably lead her to pay closer attention to this phenomenon.

At the onset of her publishing activity, in the mid-1920s, a series of Fejgin’s articles was published by the French journal Societe de Biologie. We learn from this source about Fejgin’s cooperation with Felix d’Herelle [20]. Fejgin carried out experiments on Shiga-Kruse *Bacillus* (today: *Shigella dysenteriae*), a pathogen responsible for dysentery in humans and animals. She obtained phages against these pathogens directly from d’Herelle. The author noticed that bacteria that acquired resistance to lytic agents differed in terms of biochemical and serological properties from the susceptible cultures (resistant strains were not agglutinated by polyvalent anti-Shiga serum), but most importantly, she discovered that the toxicity of resistant bacteria was reduced. Subcutaneously injected rabbits did not show signs typical for dysentery in the following hours. Noticeably, this phenomenon is being extensively investigated in recent literature, nearly a century after the publication of Fejgin’s article. Furthermore, it serves as a main argument against phage resistance developing in bacteria during phage treatment [21,22,23].

Interestingly, in her earliest work, Fejgin used the term “bacteriophage” only once when she described a phage received from d’Herelle. She was more willing to use the term “lytic agent” to describe the force leading to bacterial lysis. One explanation for this could be the fact that d’Herelle and his conception of phages were initially met with great opposition from Bordet, who was a director of the Pasteur Institute in Brussels, and his followers [24]. However, Fejgin knew how to isolate this “lytic agent” in a laboratory setting by seeding feces into a liquid medium and then filtering it [25]. She was also aware that old bacterial cultures constituted a good source of new lytic agents. Notably, following the Twort and d’Herelle articles, “bacteriophage” terminology was already in use [26,27] even before the publication of Fejgin’s first article, which indicates that back then, she might not have been entirely sure what lytic factor she was dealing with, or she may have wanted to perform her own investigations before drawing conclusions consistent with her predecessors.

One of Fejgin’s first observations toward phages was made with J. Supniewski, a Polish pharmacologist and chemist. Fejgin and Supniewski discovered that the lytic agent lysed only living bacteria and was capable of withstanding the prolonged action of ether and then retaining its vital properties intact, a feature that had not been observed in any other living structure. These revelations were published by the abovementioned Societe de Biologie in 1923 as the third article in a series [17]. Both authors suspected that the lytic agent was not able to propagate by itself and required living bacteria in order to maintain its lytic activity. It is worth remembering that such obvious conclusions had to first be discovered by researchers who knew nothing or very little about the investigated phenomena, and therefore, their significance is undeniable.

In a short article from 1925 [28], Fejgin described her investigations on bacteriophages isolated from the genus *Bacillus* causing diphtheria (today: *Corynebacterium diphtheriae*). The bacterial strain was obtained from the State Institute of Hygiene in Warsaw. Notably, the abovementioned article is probably one of the first reports suggesting toxic properties of unpurified phage lysates following bacterial lysis. Fejgin noted that filtered broth containing bacterial debris remained after bacteriophage lysis exhibited toxic properties to rabbits with clearly visible symptoms of intoxication. She continued her research on this phenomenon in the subsequent years. In an article from 1936 [29], she tested the toxicity of *Shigella* phage lysates obtained in different conditions (phages were added to 4, 20 and 40 h bacterial broth cultures). Only the latter one with the highest number of lysed bacterial cells (and correspondingly, the highest amount of toxins released by lysing bacterial cells) turned out to be lethal for intravenously injected rabbits. Interestingly, control samples deprived of bacteria through filtration in which bacterial lysis did not occur (due to phage absence) were not toxic to tested animals.

Analysis of Fejgin’s literature reveals that the previously mentioned article from 1923 [20] was not the only example of Fejgin and d’Herelle staying in contact. Another example of cooperation between both scientists comes from 1926 [30]. Fejgin and T. Epstein, who also worked with Ludwik Hirszfeld [31], received from d’Herelle a staphylococcal phage SII. Following a series of passages, Fejgin broadened its lytic spectrum until all strains from her collection became susceptible to phage activity. One could notice that such methodology based on multiple passages of phages with potential resistant bacterial hosts is still in use by phage researchers with promising outcomes. For instance, in 2021, Borges published an article entitled “How to train your bacteriophage” in PNAS, stating: “*the idea is that, by preadapting the phage to a bacterial host, the phage will experience the ways in which the host evolves resistance, and will evolve reciprocally*” [32]. Fejgin also came to another conclusion that hemolytic strains of *Staphylococcus* were resistant to phages, a fact that, according to her, was reported for the first time. Notably, this finding was cited by numerous authors in the subsequent years [33,34], including d’Herelle [35]. It was probably the first time she used the term “bacteriophage” throughout the entire manuscript to characterize the mysterious “lytic agent”. Her use of the term “bacteriophage” suggests that Fejgin was already aware of what she was investigating, most probably based on the pioneer d’Herelle’s work (at that time, she often cited d’Herelle’s methodology and his observations) and similar articles focusing on this phenomenon. Before that period, she was willing to describe the phage phenomenon as an “invisible form of bacteria in seawater” [36] or “visible microorganisms that can transform into invisible forms which can be induced experimentally” [37]. In the latter article, co-authored with M. Łazarewicz, she once again noticed that bacteria resistant to phages differed from their susceptible counterparts morphologically and biochemically. This occurrence caught her attention in the years to come. In 1933, she described it again using an example of *Shigella dysenteriae* and phage HX19. In her own words, these secondary cultures appearing after bacteriophage lysis “represent the most exciting side of the bacteriophagy phenomenon” [38].

In general, the 1930s are marked by Fejgin’s more detailed articles in which her knowledge of and experience with bacteriophages is noticeably greater. As already mentioned, in 1931, Fejgin published an article on marine microbes [19]. She described observations of other authors who noticed that sea water in which the disease took place protects marine animals from the development of symptoms. The correlation with the first observations made in the waters of the Ganges River is more than obvious. Fejgin had the opportunity to observe such marine animals (urchins) kept in diseased water. The protective effect lasted for a very long time and the examined animals died of starvation but not because of the bacterial disease. However, it must be emphasized that Hankin’s investigations carried out on samples collected from an Indian river are questioned by some authors suggesting that chemical agents dissolved in water could be responsible for bacterial inactivation [39]. It cannot be excluded that this could also happen in the case of Fejgin’s observations. In fact, our own observations of phage isolation are consistent with Hankin’s opponents and thus, it is recommended to confirm phage presence through multiple passaging to ensure the multiplication of a single phage particle from the environmental sample [40].

In 1935, Fejgin and Epstein focused on an epidemic of dysentery that took place in Poland in the city of Krzemieniec (today’s Ukraine). Although careful in making final judgments, she was aware of the potential role of phages in the course of the epidemic. The authors found active phage particles in the feces of 24 out of 25 convalescents and in 15 samples collected from 28 patients with an active disease. The authors concluded that phages, thanks to their ubiquity, constituted the most potent factor leading to bacterial variability [41]. In the same year, Fejgin and Epstein observed the coexistence of two forms of phages—one triggering the “vitreous” transformation of a single strain of *Escherichia coli*, and a lytic one causing “bacteriophagy”, as described by d’Herelle [42]. Today, we would call these phenomena lytic and lysogenic cycles.

## 5. Perception of Fejgin’s Work among Other Authors

As already mentioned, articles published by Fejgin were extensively cited during her scientific activity and, to a lesser extent, in the following years. In addition to the aforementioned d’Herelle or Coetzee, who cited her work nearly 20 years after her tragic death [16], we found several papers containing references to her work. In 1925, *Science* cited Fejgin’s observations on bacterial variability under the influence of its “lytic bacteriophage substance” [43]. Two years later, in a 700-page volume of *The Journal of Infectious Diseases* [44], Fejgin’s name was mentioned 17 times. Her work on *Bacillus dysenteriae*, *B. typhosus*, *B. proteus* was cited in terms of serological properties of bacterial isolates but also in terms of “lytic principle” and its action towards *Bacillus* strains. Almost half of the above volume was written by Philip Hadley, who used Fejgin’s, Arkwright’s and Gratia’s findings to push his cyclogenic theory on bacterial variation which was dismissed by d’Herelle. Leaving aside the validity of his theory, there is no doubt that Fejgin’s work was cited, valued and acknowledged by the most prominent scientists of her times. Notably, Hardley mentions Fejgin together with d’Herelle in one sentence as authors who were able to obtain “a lytic principle” which would be called a phage particle these days.

Bronisława Fejgin played an important role in paving the way for the advancement of research on bacteriophages and for the future generations of phage scientists. Her numerous articles from the interwar period constitute evidence of the significance of Bronisława Fejgin’s achievements. Working on a phenomenon whose nature was not fully understood certainly did not make her work easier. Despite the obstacles, she made observations that, to this day, form basic knowledge for all scientists investigating bacteriophages. In retrospect, it must be admitted that her interpretations and descriptions were surprisingly accurate. Her unique scientific intuition rarely failed her and the conclusions she drew based on her own and others’ work were balanced, without being overly optimistic, and were always supported by scientific evidence. Last but not least, her articles constitute a valuable source of knowledge about the methodology and techniques used for the isolation, filtration and purification of phages as well as media used to culture bacterial hosts during the times she lived in. Berkefeld and Chamberland filters, Soxhlet extractor, Martin broth, Chapoteaut peptone, etc., complete the picture of her daily professional life and marked directions for future generations of scientists.

## 6. Conclusions

After an in-depth analysis of Fejgin’s published work, we believe that she deserves no less attention than the phage pioneers, Twort and d’Herelle, as well as Ludwik Hirszfeld, whose work was mainly directed towards serology but, as the founder and first Director of the Institute of Immunology and Experimental Therapy, Polish Academy of Sciences in Wrocław (Poland), is inevitably associated with phage research and phage treatment of humans [45,46]. Considering the historical and political disturbances of her times, Fejgin led an incredibly active life full of discoveries, the significance of which cannot be overestimated.

Olga Amsterdamska, in her article from 1991, presents an interesting point of view that historians tend to forget about fruitful scientific activity during the pre-war period by considering it less significant compared to post-war advancements in genetics and biochemistry [47]. As a result, only a few more prominent names from that era managed to avoid being forgotten. This could be an explanation for Fejgin’s poor recognizability, so unfair yet so real. Amsterdamska describes the first half of the 20th century as a period full of tensions between biology and medicine, two disciplines that equally contributed to Fejgin’s scientific activity. Moreover, she perished during WWII, and thus, she could not continue her scientific activities during the post-war period, as many others did. She also represented the complicated ethnic and national origin of the Polish–Jewish—during post-war Poland, communists were not interested in recognizing the achievements of a pre-war “capitalist” researcher. Last, but not least, she was a woman, a rare example of a woman scientist at that time.

In conclusion, the fact that the scientific work of Bronisława Fejgin is a forgotten element of scientific history is highly unjustifiable. We sincerely hope to change it with this publication and previous articles by giving due credit to her work and achievements.
